# Further Remarks on the Tying of the Human Aorta

**Published:** 1819-05

**Authors:** Samuel Young


*For the London Medical and Physical Journal.*

### *Further Remarks on the Tying of the Human Aorta;* by SAMUEL YOUNG, Esq.

AFTER a year's lapse, some observations, under the signature of C. R., very curiously entitled "An Examination" into my Remarks on tying the Aorta, published in the Medical Journal of March 1818, have just appeared in the Number of this month. I say, observations very curiously entitled "Examination," because it is really most curious to term that an *examination*, the whole tendency of which throughout is little less than a parasitical and declamatory attempt to establish a favourite position; and that, too, by the aid of unjust insinuations and perverted statements.

Had the gentleman, C. R., confined himself simply to the question, a very few remarks would have been necessary in reply. But, although he has chosen to entangle almost every line by observations to mislead rather than to illustrate, and given to the whole a tissue of personality, I shall not be led further than just what belongs to the disentanglement of truth, to the disavowal of unworthy motives unjustly attributed, and to that which naturally grows out of the present state of the subject.

One would reasonably imagine, by the opening of the gentleman C. R.'s paper, that something strongly personal in its nature was contained in my "Remarks on tying the Aorta" as connected with Mr. Astley Cooper's published account of that operation. But what is the undeniable and unavoidable inference in the face of facts, upon reference to those Remarks, and placing the two papers together,—that is, my paper on the subject (March twelvemonth), and C. R.'s paper on the same subject, of this month?—Why, that no ground whatever exists to authorize the language C. R. makes use of in the opening as well as other parts of his paper; such, for example, as the following:—"As a former pupil of Mr. Astley Cooper, and one among his numerous admirers, I lament that such ill-founded accusations against a gentleman, whose unremitting zeal in his physiological and pathological pursuits is so well known to the world, should so long have remained without refutation; and shall endeavour to show that, of Mr. S. Young's charges, some were founded on entire ignorance of, &c."

"Ill-founded accusations—charges!" What *accusations*? what charges? By such declamatory debating-club sounds, one would really imagine that I had been making a most serious attempt upon the purity of Mr. Astley Cooper's moral character: while, on the contrary, the *reality* and *truth* of the thing is, that these *sounds* of "unfounded accusations" and "charges" are *mere sounds*, the gratuitous creation of the gentleman, C. R.; as a reference to my paper in March twelvemonth's Journal amply and incontestibly shows. In all that paper, I repeat, there is not a *line*, a *sentence*, or a *word*, that can possibly authorize the most distant allusion to any thing like "accusation," whether unfounded or not; or one *single tittle* that bears the slightest resemblance to a "charge," or the feature of a charge.

In the paper alluded to, I dissented from general conclusions, not from any individual disrespect, but simply because I honestly felt that a very serious question, connected with the interests of society, was consequently involved; and I now repeat, that I still more fully do dissent, and most solemnly protest against the operation, as a generally-received expedient for inguinal aneurism, of tying up the human aorta, as inevitably destructive: destructive, not upon mere matter of opinion, but inevitably destructive to life, not only upon the common-sense observation of things, but as established upon those detailed facts of the operation as given by Mr. Astley Cooper himself.

Now, what has the gentleman, C. R., to say against this, but simply, "as a former pupil of Mr. A. Cooper, and one of his numerous admirers," he is determined to support even his errors, if errors he have, at the expence of impartial statement, and that conduct which one gentleman owes to another. For what else, I ask, is the entire character of the paper under the initial C. R. but invidious and unnecessary personality, combined with the most culpable mis-statement.

The gentleman, C. R., is a lamentable instance of that intolerant spirit and dogmatical principle of the schools, which have opposed more difficulties to the progress of science, than even the obscurity itself in which nature lies veiled to the confined and very limited sight of human intellect. Every doctrine contrary to a favourite position, and every innovation, whatever its pretensions may otherwise be, which may militate against received or declared opinions, are by this spirit at once denounced as heresies, and the authors treated as calumniators or impostors.

Pupils too often become bigots, rather than the fair and honourable followers of their masters. Forgetting that the course of knowledge is progressive, they form their minds to a certain fixed faith, from which their own too-frequently very limited capacities will not suffer them to depart. They bask and dabble in the shallows, but never venture into the depth or height of their original.

It is here that the errors of eminent men become so truly mischievous, because they are *imitative*. In copies we find all the faults, but seldom, if ever, *the spirit*, of the master. In this feeling it was that I noticed what appeared to me palpably erroneous in Mr. Cooper's deductions on the tying of the aorta; and the gentleman, C. R., as one of his pupils, is an apt illustration of the necessity and truth of what I advanced.

The very language of the gentleman, C. R., shows how very necessary it was to "remind students that they have other duties to discharge as surgeons, as well as running about the country performing extraordinary operations, and tying-up of aortas;" for his mind is evidently typefied and possessed with this his favourite operation. He considers it what he terms "his *good fortune* to have witnessed the operation," and had actually, as appears by the date of his paper, got down as far as Bristol, no doubt to try his hand upon all that his "good fortune" might throw in his way.

The very gross personal allusions contained in the concluding paragraph of the gentleman, C. R.'s paper are unfair, as they are anonymous; but in every respect they are idle, declamatory, and (I might justly add) contemptible.

When I alluded to *eminence* and *celebrity*, I meant *that* obtained by honourable and able exertions in the pursuits of truth: I know no other eminence, nor acknowledge any other celebrity. With such a principle of mind, what can the eminence of Mr. Astley Cooper or any other individual affect me, but simply, for the good of mankind, I could wish it *more* instead of less?

Fulsome adulation at all times is odious, but, when coupled with the forfeiture of truth and justice, it becomes detestable.

As a specimen of fidelity in report and integrity of representation, the following statement of C. R. may be taken. "After the operation," says the gentleman, C. R., "the report goes on to state, that he (the patient) complained of pain not only in his head, but all over his body; his pulse was weak and fluttering; his fæces and urine passed away involuntarily; his body was covered with cold sweats, together with continual vomiting. Such is the condition of a patient in whom it is recommended by Mr. Samuel Young to open one or both jugulars."

Any who have read the report of this case, as published by Mr. Astley Cooper, and my remarks connected with the opening of one or both of the jugulars, must instantly detect a wilful omission by the gentleman, C. R., of those very facts on which my remarks entirely hinged; viz. —the evident surcharge of the blood-vessels of the head, as indicated by pain *more particularly* in his head, by the carotids beating with considerable force, and by the great anxiety expressed in the countenance. The following are the very words of the report itself, not cut, and fashioned, and garbled, with a total omission of parts, to answer the purposes of cavil and misrepresentation, but for fair practical deduction, faithfully transcribed verbatim from the notes of the case; viz.—

"27, at 7 A.M.—The report was, that he had passed a restless night; the vomiting had returned at intervals; his pulse 104, weak and fluttering; he complained of pain all over his body, more *particularly in his head*; [not as the gentleman, C. R., has got it, 'not only in his head, but all over his body,' but *more particularly* in his head;] and *the carotids beat* with considerable force. He had *great anxiety* expressed in the countenance, was very restless, and the urine dribbled from him with some degree of pain at the end of the penis." Such was the state of facts to which I generally alluded, when it appeared that there was no attempt to relieve the evidently overcharged state of the blood-vessels of the head during the life of the patient; nor any inspection as to the degree of that overcharged state, or the condition of the brain itself, after his death.

I am sorry to disturb the self-complacency of the gentleman, C. R.'s physiological statements, but his reasoning on the retrograde course of the blood happens to be the very reverse to what really does take place under the application of the ligature; and, with all submission, I beg leave to make choice of facts as they really are.

It is stated by the gentleman, C. R., in way of refutation, that, "the ligature being situated three quarters of an inch above the bifurcation of the aorta, and consequently below the superior and inferior mesenteric arteries, if the whole mass of blood had been retrograded into the vessels above the ligature, surely those arteries situated *immediately* above the impediment to the progress of the blood would have been most gorged, and we ought to have found those parts supplied by the mesenteric arteries particularly tinged with blood; but the intestinal canal was not only free from inflammation or congestion, but remarkably exsanguinous, as were the other abdominal viscera."

Now, leaving to the gentleman, C. R., to dispose of what he pleases to term "my ignorance," as well as his own physiological wisdom, just in the manner it may best suit him, (for I will not contend the point, which is the more respectable of the two,) I have only simply to state that these observations about the mesenterics, unfortunately for his wisdom, are just the very opposite to what necessarily must, and what actually did take place in the case before us, whenever a ligature is tied round an artery of considerable size; viz.—that, by the re-action, or recoil, of the blood, in consequence of the ligature, upon its *own* current, an *equipoize* must necessarily be produced to a certain extent; and, as in the instance of the tied aorta, the blood contained in the arterial tube must *consequently* become a *stationary column*, to *a certain height*, *above* the *part* where the ligature is placed. Therefore, those arteries *immediately* situated above the ligature, like the gentleman, C. R.'s unfortunate mesenterics, must necessarily be deprived of their usual supply instead of being overgorged, as the current or tide of blood from the heart cannot reach them, in consequence of the repelling force,—i. e. the ligature,—*immediately* acting beneath them.

That such is the right theory of the formation of arterial coagula under the circumstances of ligature, is too palpable to enlarge upon; and what, indeed, must necessarily be produced,—i. e. a state of rest to a certain degree above the ligature, before any coagulum could form. That this state of rest did take place above the aorta ligature is proved by the facts of the case: "a clot, of more than an inch in extent, was found to have sealed the vessel above the ligature." "All (says Mr. Astley Cooper) were gratified to observe the artery so completely shut in forty hours." The gentleman, C. R., bears evidence of the little supply of blood through the mesenteric arteries, by "the *remarkable exsanguinous* state of the intestines, as well as the other abdominal viscera." All proving the *stagnant state* of the aorta column, or at least its impaired and shortened current of blood, in consequence of the ligature; and necessarily showing the increased quantity that must inevitably be driven upwards through the carotids; and which "their strong beatings," with the other head symptoms of the patient, as already noticed in the case before us, most unequivocally establish. In short, without previous very copious bleedings, I feel convinced in my own mind that apoplexy would be the almost immediate result after passing the ligature round the aorta, in a man of any moderate state of health. In the individual instance before us, the man was almost drained to death by previous bleedings, and yet the symptoms evidently proved the very surcharged state of the vessels of the head after the ligature was made.

The observations of the gentleman, C. R., upon tying the femoral artery, are really so very vague and frivolous, so entirely from the point in question, that it is impossible to afford them the slightest serious attention. What have the known and familiar results from tying-up the femoral artery to do in comparison with passing a ligature upon the main aorta itself, where by necessity, as already shown, the carotids, with the subclavians, must be the only important outlets for the repelled current of blood; the portion between the curvature of the aortal arch and the ligature being, in consequence of the ligature, in a gorged and almost stagnant state?

"I would ask (says the gentleman, C. R., in reference to the state of the aneurismal limb,) what is the distinction between a limb assuming a cold and livid state, and the incipient stage of gangrene?" This, indeed, is asking a question with a vengeance. The difference, I humbly should imagine, is precisely that which exists between a thing that is and a thing that is not; an object expected, and an object effected; an accident that may take place, and one that has already happened; the state of a ship that may very possibly sink, and one already sunk; in short, just the difference between the existence of a low degree of animal life, and the absolute death of the part: but certainly not that palpable distinction which exists between common sense and the gentleman C. R.'s question.

However truly absurd such a question may be considered by more reflecting minds, yet a very different estimation is held of it by the gentleman, C. R. He evidently not only considers it a most sapient, sound, and solid observation, but particularly happy and conclusive on the subject; for, evidently with a feeling of the most satisfied triumph, he immediately adds, "Mr. Young must have very extraordinary notions of the animal economy, if he can suppose that nature will not take cognizance of the circulation in a whole limb being permanently impeded, and can refuse to consider such circumstances as sufficient to derange, and at length destroy, the functions of the whole machine."

To such an observation I can only rejoin by remarking, that *the notions of the animal economy* must be still *more extraordinary* with him who, after *a ligature* has been passed around the *human aorta*, can *overlook* such a circumstance, and attribute the *cause* of death merely to a deficient circulation in an aneurismal limb, *the reported continued heat of which*, although six or seven degrees *under* that of the opposite member, was at 87½.*

The labours of the gentleman C. R.'s paper end here, and further on its frailties I have only to assure the Journal, its readers, and the gentleman, C. R., that I will never again trouble them with a single syllable.

A few observations, however, since "the game's a-foot," may properly follow an apparent contradiction, which

---

* The evidently distinct state of things which must necessarily exist by the slow and progressive diminution, by disease, of the aortal artery, in opposition to the *sudden* and *instantaneous* check to the *whole* current and *full impetus* of the blood in *such an artery* where a *ligature* is made, appeared to my mind so very obvious, that I felt to dilate on such palpable distinctions, in the present paper, as well as in those few cursory remarks made at the instant, which formed my paper of March twelvemonth, would only be a most unnecessary waste of time and occupation of place.

In a physiological point of view, to make such a comparison, and draw conclusions in favour of the aortal ligature, because that artery has been progressively diminished, or obliterated in parts, by disease, would be no other than to draw favourable results in consequence of *sudden* injuries of the brain; because, forsooth, nearly the whole of that viscus has been changed, obliterated, or removed, by the various and slow advances of variously-diseased accumulations or morbid growths and actions.

would seem to exist in the practical statement and conclusions of Mr. Astley Cooper himself on the present subject.

"At nine o'clock the same evening," observes Mr. Cooper (Essays, p. 118), "I saw him, and found him in so reduced a state, that he could not survive another hæmorrhage, with which he was every moment threatened: yet, still anxious to avoid opening the abdomen to secure the aorta near to its bifurcation, I determined to ascertain whether it was practicable to pass a ligature around the artery from within the aneurismal sac; for I was of opinion that, if the artery had given way near the centre of the sac, as it usually does in aneurism, I might compress it with my finger, and pass a thread around it. With this view, I made a small incision upon the aneurism, &c."

This attempt of Mr. Cooper's failed, in consequence of the entire expansion or loss of the artery in the aneurismal sac. This attempt was made, of course, *before* the great operation of tying the aorta. However, after this last operation,—i. e. the tying of the aorta, and reasoning on the state of the aneurismal sac, Mr. Cooper then immediately draws the following conclusion:—"In an aneurism, therefore, similarly situated, the ligature (meaning the ligature, of course, around the aorta,) must be applied *before* the swelling has acquired any very considerable magnitude."

Here a most serious predicament is evidently created: the author stands most fatally opposed against himself. Either the attempt to tie the artery in the aneurismal sac was right, and not an unnecessary precaution, or the practical conclusion for passing the ligature round the aorta at an *early period*, "before the aneurismal swelling has acquired any very considerable magnitude," must be wrong; or, if right, then it must necessarily stand fatally opposed and in open contradiction to all the precaution and waiting previously evinced and insisted upon by Mr. Astley Cooper.

As far as authority goes, wherein, then, lies the right practice in this case? On the one hand, we are told, by no mean authority, that the passing of the ligature around the aorta must be one of our early attempts; I say *early*, because we are directed to do it *before* the aneurism (or such or similar aneurisms as the one now in question) gets to any size; or, which is the same thing, "*before* the swelling has acquired any very considerable magnitude." And yet, on the other hand, but a few short pages preceding, as well as in the preamble to this aortal case, this same authority—and no mean authority, let it be repeated and re-echoed,—shows, by his great disinclination to open the abdomen, in having recourse to almost every expedient, (although, as he himself states, another bleeding, which was momentarily expected, would have inevitably destroyed the patient,) the great risks and eminent hazard, even in his own mind, that must necessarily be run when such an operation is concluded upon as that of tying the human aorta: and yet, after all this, as already seen, in a few short pages, it is laid down by this same authority (without the necessity of any previous attempt or compromise being shown or hinted at) as a general rule of practice—that the aorta must be tied at the early stages of the disease; i. e.—" *before* the swelling has acquired any very considerable magnitude."

Now, I ask, in this state of palpable contradiction, where is the true line of practice to be found? If this is cavil, then I confess I know nothing of the principle of right argument, and give up all pretensions to fair and honourable discussion: but, on the contrary, I feel convinced that what has been here stated is a fair exposure of facts, the fallaciousness of which has gone to the countenance and establishment of that which would prove, if adopted, a most fatal practice.

Mr. Cooper, however, has taken his stand upon this case (whatever erroneous conclusions may have been drawn upon its issue) on the surest basis of right policy—" the do as you would be done by;" a sound, however awkward to some professional ears, which carries with it true wisdom, and is that which raises our profession superior to any in useful and honourable pursuit; as its absence debases it to the level of a mercenary traffic in human calamity.

" In the performance of our duty," says Mr. Astley Cooper, " one feeling should direct us: the case we should consider as our own, and we should ask ourselves whether, placed under similar circumstances, we should choose to submit to the pain and danger we are about to inflict." I repeat this great line of conduct as here detailed by the author, (a principle the most important through life, but most particularly so in connexion with our own profession,) because it is far more gratifying to dwell upon such manly exposition of the mind, than upon any error of judgment that may be formed upon it. This, indeed, is a precept worthy in us all of honourable and emulative imitation, and should sink deep particularly in the mind of the student, who but too frequently, after passing the necessary career of professional initiation, thinks, in the ardour of his pursuits, that, with a scalpel in his hand, he grasps the sceptre of surgery.

It is on this great principle of common justice and duty, which ought to guide all our actions, that I ask a reconsideration of this sentence—"The aorta must be tied before the swelling has acquired any very considerable magnitude." Of this sentence, whether it be not founded on an error in judgment, as already insisted upon, I ask a fair, impartial, and ample re-consideration; at least, before it shall be so passed as a rule of practice.

There were two expedients, Mr. Astley Cooper tells us, tried before he thought of venturing upon opening the abdomen: the one, his endeavour to tie the artery in the sac; the other, the application of pressure upon the aneurismal tumor. Are such attempts, in similar cases, entirely to be deserted?

"Before the swelling has acquired any very considerable magnitude," instead of tying the aorta, how is it that Mr. Cooper has forgotten to tell us to tie the artery in the sac? There is surely a much better chance of meeting with the artery in this early stage than when Mr. Cooper attempted to tie it: yet this attempt was made in the very last extremity of the disease, before he thought of such a thing as the opening of a man's abdomen to get at the aorta itself: but now, in the earlier stages of the disease, when we say,—that is, when the fiat is passed—that the aorta must be tied,—then, indeed, it is not fitting to say a word about the artery in the sac, though the possibility of finding it in the one instance is here changed into every probability.

The fact is, we are *here* dazzled with a great operation, and we do not see things quite so clear as might be expected.

The other expedient adopted was that of pressure, although most improperly managed, as the names of the materials and the method of their application, with the consequent slough or ulceration of the integument, but too evidently show: such, for example, as a cushion bound down on the part, together with the use of the tourniquet, instead of uniform rolling of the limb and pelvis, and the *gradual* application of compress, until the aneurism at length is specifically commanded; and, where the aneurismal integument is much diseased and distended, the pressure, in the first instance, should be *general support only* until the parts are recovered and gained upon; for here the great object is to *preserve*, not to *slough* and *ulcerate*, as such things as the pad or cushion, with the tourniquet used by Mr. Cooper, most inevitably will produce; without, indeed, the integument is in a perfectly sound state, and but slightly distended.

Pressure, however, is surely most worthy of notice and due consideration, before such an extraordinary effort is made as the tying of the human aorta; since pressure has been *known* to have effected the obliteration and cure of inguinal aneurism. Yet it is said, without the slightest consideration of these facts, that the aorta must be tied in the earlier stages of the disease,—that is, " before the swelling has acquired any very considerable magnitude;" in which state the chances are that the disease may be obliterated by pressure, or the artery very probably secured, in the sac itself.

I shall purposely avoid entering further into the detail of any method of practice for the disease in question, that the great point in debate may not be compromised. On the individual case and the operation, as given by Mr. Astley Cooper, I have already, a twelvemonth back, expressed my entire conviction, as grounded upon the facts of the case,—viz. that, viewed individually, and at the time it was performed, the tying of the aorta was the only thing left among the *last snatches* of *chance* to save the life of the patient; and that the operation was as boldly conceived as it was admirably performed. But further than this I do not go.

I dissent from the conclusions drawn from the facts of the case; and more particularly from the very summary mode of summing up the evidence, for the purpose of establishing an extended rule of practice from this solitary, and in many instances, inapplicable case,—viz. " that the aorta must be tied before the disease has acquired any very considerable magnitude."

It is on this point, upon the evidence and authority of facts, that I enter my most decided protest: on this ground, individually, I earnestly entreat a re-consideration of all the facts of the case. But more especially is it, viewing the relation betwixt the author and a believing and confiding public, that I request not only a re-consideration, but also a fair and full recantation, if an error of judgment should be found to exist; or, at least, if no such error even should be found, a further exposition of facts to authorize the adoption and extension of so extraordinary a practice.

*Gerard-street;*

*March* 15, 1819.

